# M1 macrophage predicted efficacy of neoadjuvant camrelizumab combined with chemotherapy vs chemotherapy alone for locally advanced ESCC: A pilot study

**DOI:** 10.3389/fonc.2023.1139990

**Published:** 2023-03-10

**Authors:** Shu Wang, Guanghui Xu, Mengbin Li, Jiyang Zheng, Yuhao Wang, Xiangying Feng, Jialin Luo, Shibo Wang, Huan Liu, Weiming Duan, Hushan Zhang, Depei Huang, Feilong Zhao, Yongzhan Nie, Jianjun Yang

**Affiliations:** ^1^ State Key Laboratory of Cancer Biology, National Clinical Research Center for Digestive Diseases and Xi-jing Hospital of Digestive Diseases, Fourth Military Medical University, Xi’an, China; ^2^ Department of Digestive Surgery, Xi Jing Hospital, The Fourth Military Medical University, Xi’an, China; ^3^ The Medical Department, 3D Medicines Inc., Shanghai, China

**Keywords:** esophageal squamous cell carcinoma, neoadjuvant therapy, camrelizumab, M1 macrophage, immune microenvironment

## Abstract

**Introduction:**

The efficacy and safety of immunotherapy have been widely recognized in gastrointestinal-related cancers. However, the efficacy of neoadjuvant camrelizumab for locally advanced esophageal squamous cell carcinoma (ESCC) has not been firmly established. This study compared the efficacy of camrelizumab in combination with neoadjuvant DCF (docetaxel, cisplatin and fluorouracil), with DCF alone for ESCC, and exploring biomarkers related to immune infiltration of the ESCC immunotherapy response.

**Methods:**

We enrolled and randomly assigned patients with stage II-IVa ESCC to two study treatments: camrelizumab combined with docetaxel, cisplatin and fluorouracil (DCF) regimen and DCF regimen alone. The tissue for multiplex immunofluorescence (mIF) was obtained before and after neoadjuvant therapy. The Response Evaluation Criteria in Solid Tumors RECIST Version 1.1 (RECIST 1.1) and Tumor Regression Grade (TRG) was used to evaluate efficacy.

**Results:**

A total of 30 patients were enrolled in the study. Following neoadjuvant camrelizumab, the objective response rate (ORR) and the disease control rate (DCR) were 46.7% (7/15) and 95.7% (14/15), respectively. No patients reported complete remission, while ORR and DCR in the chemotherapy group were 26.7% (4/15) and 86.7% (13/15), respectively. R0 resection after neoadjuvant treatment was achieved in 3 out of 15 patients in the combined group and in all patients (15/15) in the chemotherapy group. In the combined group, M1-type tumor-associated macrophages and CD56dim NK cells were more abundant in responders than in non-responders (p < 0.05). A higher M1/M2 ratio was observed in responders (p < 0.05). With respect to the NGS, among the copy number amplified genes, the 11q13 amplicon (CCND1/FGF19/FGF4/FGF3) showed the highest frequency (47%, 7/15).

**Conclusions:**

Neoadjuvant camrelizumab combined with chemotherapy improved ORR in locally advanced ESCC. M1-type tumor-associated macrophages and CD56dim NK cells might be utilized to predict camrelizumab efficacy.

## 1 Introduction

The esophageal squamous cell carcinoma (ESCC) is a high-mortality cancer with complex etiology. In China, 252,500 new cases and 193,900 deaths have been reported during 2016 (1). The five-year survival rate is poor, ranging from 23% to 38% (2–4). Regarding the non-metastatic ESCC, neoadjuvant chemoradiation followed by esophagectomy is the recommended treatment strategy by current National Comprehensive Cancer Network guidelines (5). Despite neoadjuvant chemoradiation and surgery, the 3-year survival rate of non-metastatic ESCC is 60%. A Real world, multicenter JCOG1109 study conducted by Satoru Matsuda evaluated neoadjuvant DCF versus CF in ESCC (6). Survival analysis showed that DCF group OS was significantly longer than CF group, but he recurrence free survival rate following resection is 50% only at 2 years in DCF group (6). The ESCORT phase 3 trial investigated the efficacy and safety of camrelizumab plus chemotherapy vs placebo plus chemotherapy as a first-line treatment in advanced or metastatic ESCC. The study showed that camrelizumab prolonged overall survival and improved response rates (7). A pilot study suggested good efficacy and acceptable tolerance of neoadjuvant camrelizumab plus chemotherapy in locally advanced ESCC (7). Another Chinese study reported similar findings (8). A single-center, single arm study using neoadjuvant immunotherapy plus chemotherapy in locally advanced ESCC evaluated the predictive power of T cell cytotoxicity in the tumor microenvironment (9, 10). An increasing number of studies found that neoadjuvant immunotherapy is beneficial in patients with ESCC. However, biomarkers associated with efficacy have not been firmly recognized. In addition, chemotherapy has never been used as control treatment.

The use of immunotherapy has been one of the most promising developments in ESCC. A positive response to immunotherapy usually relies on dynamic interactions between tumor cells and immunomodulators inside the tumor microenvironment (11). Neoadjuvant immunotherapy may shrink the tumor, downstage nodal status and increase the likelihood of margin-negative resection (12). This pilot study aimed at investigating prospectively the efficacy and safety of neoadjuvant camrelizumab combined with chemotherapy vs. chemotherapy alone for locally advanced ESCC. Additionally, we explored potential tumor immune microenvironment biomarkers predicting camrelizumab efficacy.

## 2 Materials and methods

### 2.1 Patient characteristics

This was a single-centered prospective, randomized controlled trial performed in patients with stage II–IVa ESCC admitted to the Xijing Hospital of Digestive Diseases, Fourth Military Medical University from Mar 2018 to Apr 2021. The enrollment criteria included: (1) Patients must be aged 18–75 years; (2) Patients with esophageal squamous cell carcinoma diagnosed by histology; (3) The primary treatment of patients with ESCC had no previous operation;(4) According to eighth edition of TNM stages, patients were divided into T2N0~1M0 or T3~4aN1~2M0 esophageal squamous cell carcinoma; (5) ECOG PS score is 0-1; (6) Estimated lifetime ≥ 3 months; (7) At least one measurable lesion (CT examination diameter ≥ 1cm, other examination methods >= 2cm); (8) Patients who can be received liquid diet at least; no signs of the before esophageal perforation; Without distant metastasis; The patients also can tolerate the operation; (9) The function of main organs is normal. The exclusion criteria were: (1)age over 75 years; (2) with severe cardiac and pulmonary dysfunction or various diseases that were not tolerable to intravenous chemotherapy; (3) there were immune diseases or were unsuitable for immunotherapy in the active period of hepatitis B; (4) cervical esophageal cancer, other malignant tumors or multiple sources of malignant tumors were diagnosed within 5 years; (5) without chemotherapy, surgery or traditional Chinese medicine treatment before enrollment; (6) related clinical data was incomplete. Thirty patients who met the inclusion criteria were randomly divided into the combination group (15 cases) and the simple chemotherapy group (15 cases).

The study was done in accordance with the Declaration of Helsinki and the Good Clinical Practice Guideline. The protocol and all amendments were approved by the institutional review board or independent ethics committee of each study site. All patients provided written informed consent.

### 2.2 Patient treatment

Neoadjuvant immunotherapy combined chemotherapy (combined group): (I) camrelizumab 200 mg, intravenous infusion, d1; (II) docetaxel 60 mg/m2 + oxaliplatin 85 mg/m2 intravenous drip, d1 + 5-FU 700 mg/m2/day as a continuous intravenous infusion for 5 days (days 1–5) every 3 weeks. Neoadjuvant chemotherapy (simple chemotherapy group): (I) docetaxel 60mg/m2 + oxaliplatin 85 mg/m2, intravenous drip, d1 + 5-FU 700 mg/m2/day as a continuous intravenous infusion for 5 days (days 1–5) every 3 weeks. Assignments were based on a predetermined randomization scheme (using a random number table) in a 1:1 (n = 15 patients per group) allocation ratio. All patients were treated for 21 days as a cycle, and surgery was performed 4–6 weeks after three cycles at 3 patients in combined group, 15 patients in chemotherapy group. Before the operation, the whole-body imaging examination and evaluation were performed, including neck, chest, upper abdomen contrast-enhanced CT, and upper gastrointestinal angiography.

### 2.3 Evaluation of response

Postoperative pathological examination was needed to determine whether the cutting edge was negative. According to RECIST version 1.1, the response of the tumor was examined after Neoadjuvant therapy by the researcher. Most common forms of medical exams conducted in this study were radiographic tests, such as CT and MRI. The outcome was divided into complete response (CR), partial response (PR), stable disease (SD), and progressive disease (PD). The proportion of CR + PR was objective response rate (ORR), and the proportion of CR + PR + SD was disease control rate (DCR). Adverse events were any adverse clinical events that occurred in treatment, and were evaluated according to CTCAE 4.0.

The tumor regression degree was evaluated by the proportion of scar and residual tumor, and it was grading into 5 degrees according to Mandard’s TRG system (13): grade 1 is no residual tumor, grade 2 is residual tumor <10%, grade3 is residual tumor 10–50%, grade 4 is residual tumor >50%, grade 5 is no regression. Endoscopic biopsy was used to evaluate the effectiveness of patients in the combined group who did not have surgery.

### 2.4 NGS and TIME

Tumor samples were collected from core-needle biopsies at baseline before neoadjuvant and from the surgical specimen obtained at the time of surgery after neoadjuvant in both groups(combined group: n = 18, chemothery group: n = 30). Tumor samples were collected from core-needle biopsies in 12 patients after neoadjuvant in without surgery combined groups(n = 12). These freshly cut tissue sections were used for analysis of the tumor microenvironment multiplex fluorescence immunohistochemistry (mIHC) analysis. Tumor samples for NGS were collected from core-needle biopsies at baseline before neoadjuvant(n = 15).

The fresh tissue samples were obtained from a gastroscopic biopsy for next generation sequencing in combined group. Combined group provides peripheral blood samples which were analyzed for germline mutations. Paraffin embedding and formalin fixation were used to fix the specimens for testing. Genomic DNA was processed by 3DMed Clinical Laboratory Inc a College of American Pathologists (CAP)-accredited and Clinical Laboratory Improvement Amendments (CLIA)-certified laboratory for NGS on Illumina Nextseq 10000 to >10000X coverage.

Investigation of the TIME was performed by 3D Medicines, Inc. PD-L1 expression was assessed using the PD-L1 IHC 22C3 pharmDx assay (Agilent Technologies, CA, USA) and was expressed as combined positive score (CPS) by dividing the number of PD-L1-stained tumor and immune cells with the total number of viable tumor cells and multiplying by 100. Tumor parenchyma and stroma were differentiated by pan-CK staining (The pan-CK positive area with DAPI staining was defined as tumor region, and the pan-CK negative area with DAPI staining was defined as stroma region). The quantities of various cell populations were expressed as the number of stained cells per square millimeter (cells/mm2) and as the percentage of positively stained cells in all nucleated cells (%). The consistency and percentage of 19 biomarkers in tumor and stroma regions were figured out by detecting signal channel or multiple-channel, namely PD-L1^+^, PD-1^+^, pan-CK^+^, PD-L1^+^&pan-CK^+^, PD-1^+^&pan-CK^+^, PD-1^+^&PD-L1^+^&pan-CK^+^, CD3^+^, CD8^+^, PD-1^+^&CD8^+^, CD4^+^, FoxP3^+^, CD4^+^&FoxP3^+^(Treg), CD20(B cell), CD56(NK cell), PD-L1^+^&CD68, CD68^+^CD163^-^(M1 macrophage), and CD68^+^CD163^+^(M2 macrophage). Multiplex immunofluorescence (mIF) staining was conducted using the PANO 7-plex IHC kit following manufacturer’s instructions (Panovue, Beijing, China). Multiplex stained slides were scanned using a Mantra system (PerkinElmer, MA, USA) configured to capture fluorescent spectra at 20 nm wavelength intervals from 420 nm to 720 nm with a fixed exposure time and an absolute magnification of ×200 and ×100. All scans for each slide were then superimposed to obtain a single image. Images of unstained and monoplex stained slides were used to extract tissue autofluorescence and the spectrum of each fluorophore, respectively. They were also used to create a spectral library required for multispectral unmixing using the inForm Image Analysis software v.2.4 (PerkinElmer, MA, USA). Slide images were reconstructed without autofluorescence using this spectral library. The quantity of immune cells was expressed as the number of stained cells per square millimeter.

### 2.5 Statistical analysis

Huiyan Luo et al. reported that the median progression-free survival was 6.9 months in the camrelizumab-chemotherapy (paclitaxel and cisplatin) group vs 5.6 months in the placebo-chemotherapy (paclitaxel and cisplatin) group (HR for progression or death, 0.56 [95% CI, 0.46-0.68] (14). On the basis of evidence from these studies, we predicted that 1-year PFS rate would be 28% in the neoadjuvant camrelizumab combined with DCF group and 7% in the DCF group. The randomization ratio is 1:1. Therefore, for the comparison of neoadjuvant camrelizumab combined with DCF with neoadjuvant DCF, a sample size of 33 patients per group was needed for 80% power with a two-sided significance level of 0.05. To compensate for a 15% drop out, an optimum sample size for this clinical trial was 72 patients, which came to 36 patients in each group. The current interim analysis was based on population after the inclusion of 30 patients.

Sample size calculations were performed in PASS 11.0 software. R (version 4.0.2). software was used for statistical analysis. The continuous variables per the normal distribution were expressed by means ± standard deviation, and a t-test was used. The classified variables were expressed by cases (%), and the chi-square test or Fisher exact probability method was used. The ordered variables and the continuous variables not in accordance with the normal distribution were tested by rank-sum test. P<0.05 was considered statistically significant.

## 3 Results

We enrolled 30 patients between March 2018 and April 2021. The baseline characteristics were similar between the study groups (**Table 1**). There was no significant difference between the two groups regarding age, gender, history of smoking, site of primary tumor, grading and staging (p > 0.05).

**Table 1:** Clinical characteristic of squamous cell carcinoma before chemotherapy.

Characteristics	No. (%)		P value
	Camrelizumab + DCF	DCF	
Age at diagnosis, years			0.8616
Mean ± S^a^	61 ± 7.8	63 ± 7.6	
Median [range]	64 [46–73]	64 [49–74]	
Gender			0.4828
Male	13 (86.7)	15 (100)	
Female	2 (13.3)	0 (0)	
History of smoking			
Former or current	9 (60.0)	8 (53.3)	0.9999
Never	6 (40.0)	7 (46.7)	
Site of primary tumor			0.225
Upper thoracic	1 (6.7)	1 (6.7)	
Middle thoracic	11 (73.3)	13 (86.6)	
Lower thoracic	3 (20.0)	1 (6.7)	
Histologic grade			
Well differentiated	1 (6.7)	5 (33.3)	
Moderately differentiated	9 (60.0)	8 (53.3)	
Poorly differentiated	5 (33.3)	2 (13.3)	
T stage			0.2588
T2	3(20.0)	1(6.7)	
T3	6(40.0)	11(73.3)	
T4	6(40.0)	3(20.0)	
Clinical staging			0.3666
II	1 (6.7)	2 (13.3)	
III	10 (66.6)	9 (60.0)	
IVA	1 (6.7)	3 (20.0)	
NA^b^	3 (20.0)	1 (6.7)	

^a^, standard deviation; ^b^, not available.

### 3.1 Clinical and pathological findings

There was no significant difference between the two groups (**Table 2**). In the combined group, two patients showed a complete response (13.3%), five patients showed a partial response (33.3%), seven patients reported a stable disease (46.7%) and a single patient reported a progressive disease (6.7%). The objective response rate (ORR) and the disease control rate (DCR) were 46.7% (7/15) and 93.3% (14/15), respectively. In the chemotherapy group, no patients reported a complete response. Four patients reported a partial response (26.7%), two patients reported a progressive disease (13.3%) and nine patients reported a stable disease (60.0%). The ORR and DCR were 26.7% (4/15) and 86.7% (13/15), respectively.

**Table 2:** Comparison of two groups after neoadjuvant therapy.

Group	CR (%)	PR (%)	SD (%)	PD (%)	ORR (%)	DCR (%)
Combined group	2 (13.3)	5 (33.3)	7 (46.7)	1 (6.7)	46.7	93.3
Chemotherapy group	0	4 (26.7)	9 (60.0)	2 (13.3)	26.7	86.7

The classified variables were expressed by cases (%), PR, partial response; SD, stable disease; PD, progressive disease; ORR, objective response rate; DCR, disease control rate.

After three courses of neoadjuvant therapy, the surgical resection of the tumor was performed within 3 to 4 weeks. In the combined group, twelve patients did not undergo any surgery. Four patients were unable to undergo surgery due to the progression of lesions or serious adverse events, while two patients reported financial difficulties. Six patients declined surgery because the subjective symptoms disappeared. A follow-up gastroscopy showed no residual viable tumor cells in these six patients. In the chemotherapy group, all patients underwent surgery.

### 3.2 Responders and non-responders

In the combined group, 2 patients showed a complete response (13.3%), 5 patients showed a partial response (33.3%), 7 patients reported a stable disease (46.7%), and one patient showed disease progression (6.7%). Twelve patients did not undergo any surgery. Three of them showed tumor cells in biopsy tissues and were considered non-responders. The other nine patients showed no signs of tumor cells and were considered responders. The other three patients were identified as non-responders. After surgery, they achieved a tumor regression grading of 3, 4 or 5. In the chemotherapy group, four patients with partial response were considered responders. Other nine patients with stable disease and two patients with progressive disease were considered non-responders.

### 3.3 Changes in the tumor immune microenvironment

**Figure 1:**
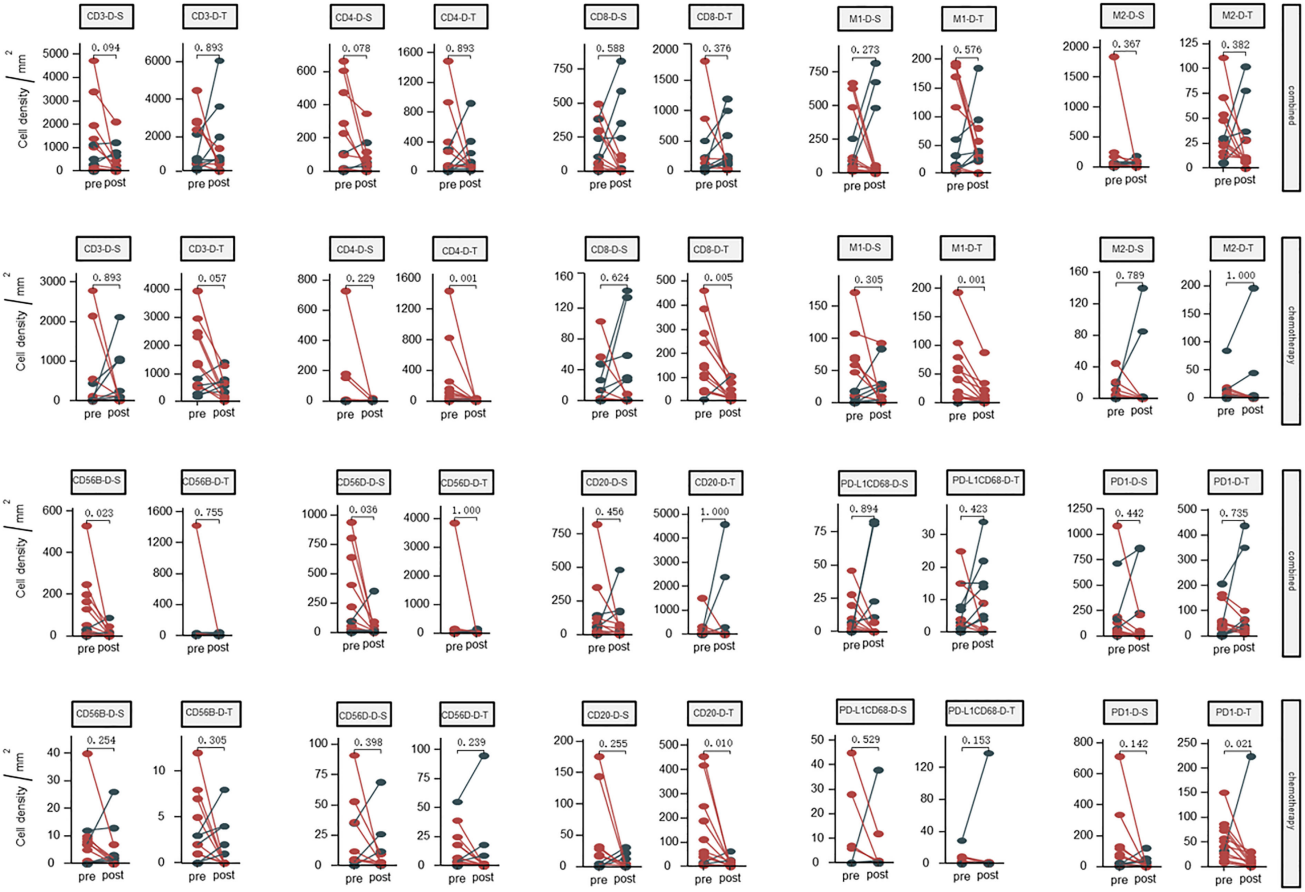
Dynamic changes of densities in the tumor microenvironment (tumor and stromal regions) before and after neoadjuvant therapy; Combined, immunochemotherapy; Pre, pre- neoadjuvanttherapy; Post, post- neoadjuvanttherapy; D, density; S, stroma region; T, tumor region; M1, CD68^+^CD163^-^ macrophage; M2, CD68^+^CD163^+^ macrophage; CD56B, CD56 bright NK cell; CD56D, CD56 dim NK cell; P < 0.05, Significant difference.

The results are presented in **Figure 1**. No significant differences were found regarding the density of CD3^+^ T cells, CD4^+^ T cells, CD8^+^ T cells, M1 tumor-associated macrophages, CD20^+^ B cells and T cells expressing PD1 before and after neoadjuvant therapy in the combined group. The CD56dim cells were significantly decreased in the stromal zone in the combined group, but not in the tumor zone in the chemotherapy group.

### 3.4 Immune biomarkers of responders and non-responders

**Figure 2:**
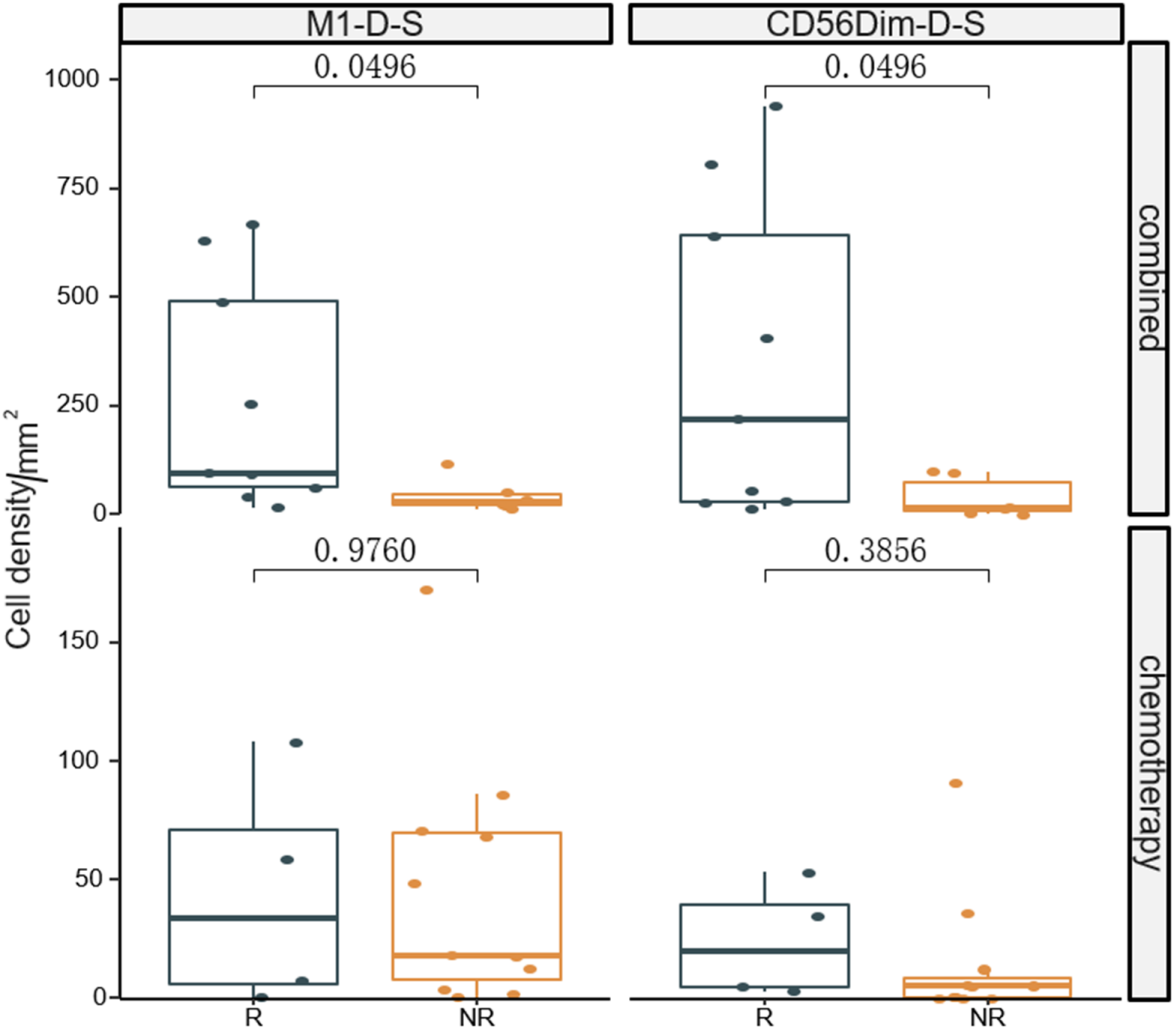
Comparison of densities of M1 macrophages and CD56^dim^ NK cells in the stromal zone between responders and non-responders the significance level of M1 macrophages and CD56dim NK cells from baseline between the two groups were compared. R, responder; nR, non-responder; Combined, immunochemotherapy; D, density; S, stroma region; M1, CD68^+^CD163^-^ macrophage; M2, CD68^+^CD163^+^ macrophage; P < 0.05, Significant difference.

**Figure 3:**
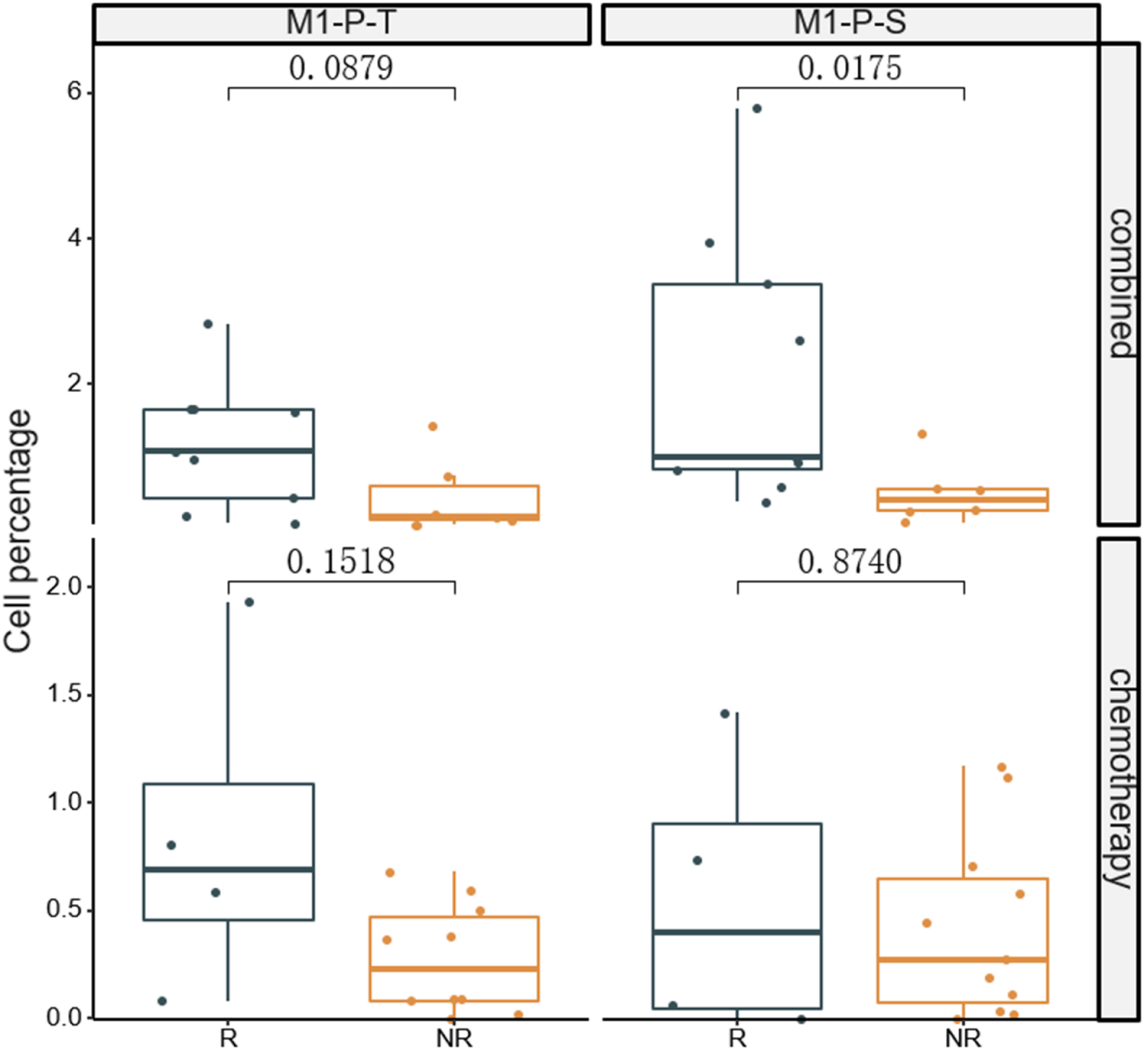
Comparison of percentages of M1 macrophages in tumor (T) and stromal (S) regions between responders and non-responders.The significance percentage of M1 macrophages from baseline between the two groups were compared. R, responder; nR, non-responder; Combined, immunochemotherapy; P, percentage; S, stroma region; T, tumor region; M1, CD68+CD163- macrophage; P < 0.05, Significant difference.

**Figure 4:**
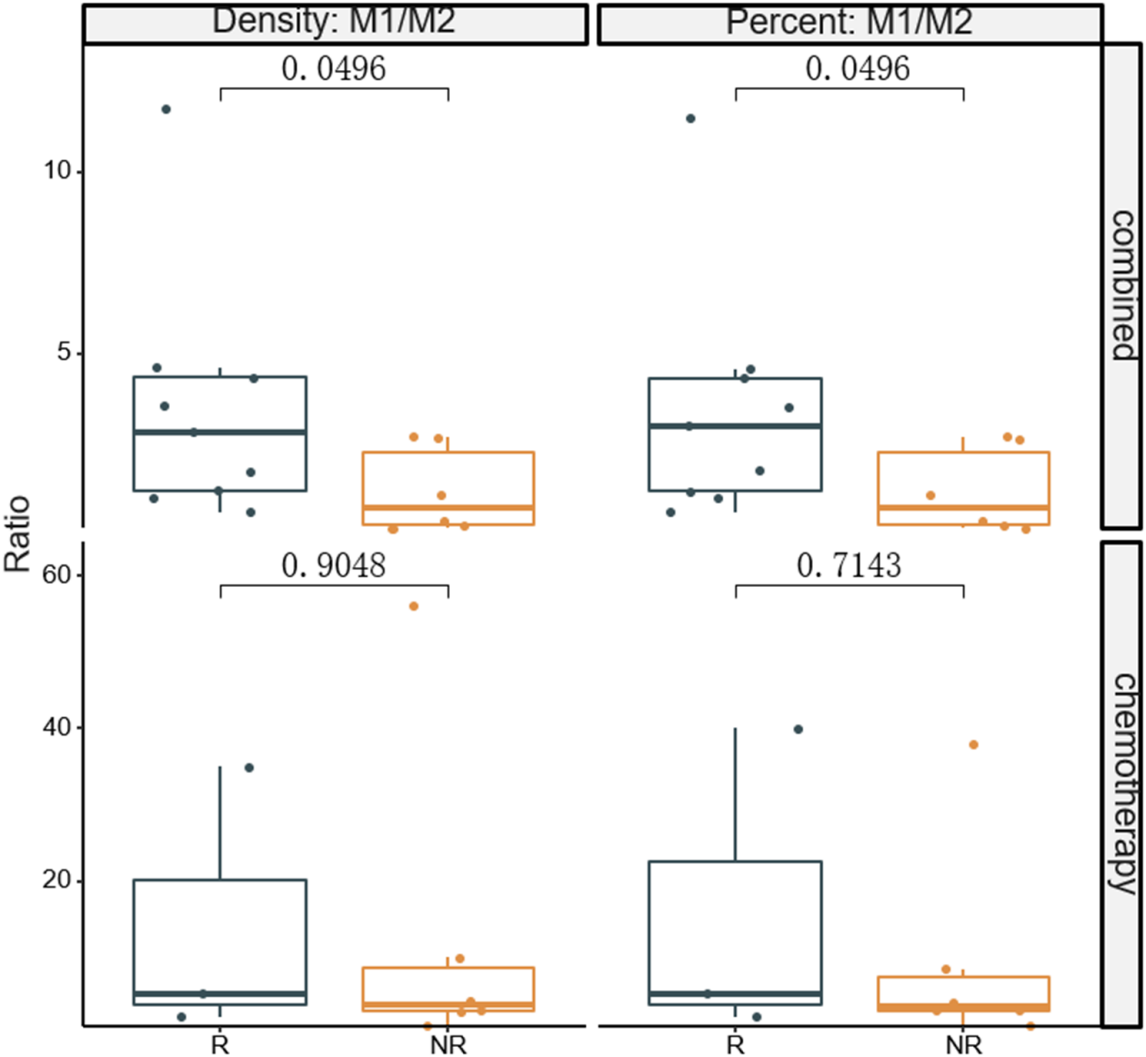
Comparison of densities and percentages of M1/M2 ratio in the tumor zone between responders and non-responders. The significance level of M1/M2 from baseline between the two groups were compared. R, responder; nR, non-responder; Combined, immunochemotherapy; D, density; S, stroma region; M1, CD68+CD163- macrophage; M2, CD68+CD163+ macrophage; P < 0.05, Significant difference.

**Figure 5:**
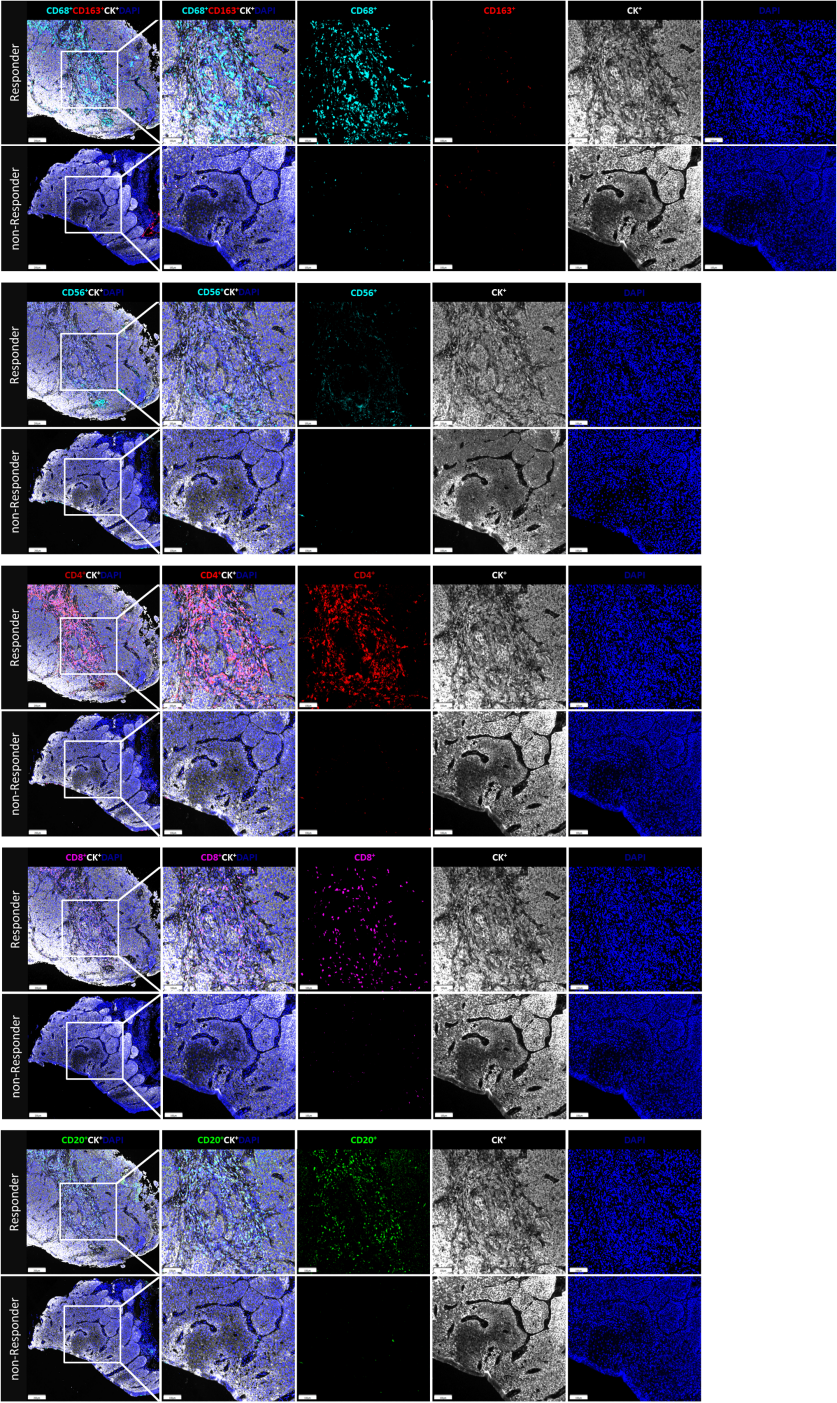
The immune cell biomarkers of tumor tissue samples from representative patients brfore treated with neoadjuvant immunochemotherapy. In responder and nonresponder, tissues from ESCC patients were stained with multiplex immunofluorescence for immune cell biomarkers, indicated by distinct colors. The density and percentage of CD68^+^CD163^-^, CD68^+^CD163^-^, CD56^+^, CD4^+^, CD8^+^, and CD20^+^ immune cells in the tumor center or stroma were analyzed from top to bottom. Representative images showing the multiplex immunofluorescence staining for identifying the immune cell subsets in the tumor immune microenvironment; Tissue-1: CD68, cyan, opal 480; CD163, red, opal 620; CD8, magenta, opal 690; Tissue-2: CD56, cyan, opal 480; CD4, opal 520, red; CD20, Green, opal 620; Two tissue samples were utilized for analysis; Multiplex stained slides were scanned using a Vectra Polaris Quantitative Pathology Imaging System (Akoya Biosciences) at 20 nm wavelength intervals from 440 nm to 780 nm. All scans for each slide were then superimposed to obtain a single image.

The fluorescent multiplex immunohistochemical analysis investigated the tumor immune microenvironment. Densities and percentages of CD8^+^ T cells, CD4^+^ T cells, FoxP3^+^ T cells, CD20^+^ B cells, M1 and M2 tumor-associated macrophages and NK cells (CD56dim and CD56bright) were quantified. The M1 tumor-associated macrophages and CD56dim NK cells were more abundant in the stromal zone of responders, compared to non-responders (p < 0.05) (**Figure 2** and **Figure 3**). No differences were observed in the chemotherapy group (p = 0.976 and p = 0.385). The M1/M2 ratio in the tumor zone was significantly higher in responders than in non-responders (p < 0.05) (**Figure 4**). No significant difference was observed in the chemotherapy group. Representative images of combined group and chemotherapy group mIHC images showed in **Figure 5**. The density of M1 macrophages, CD56^+^, CD4^+^, CD8^+^, and CD20^+^ immune cells in the responder were higher than nonresponder.

### 3.5 Genomic analysis

**Figure 6:**
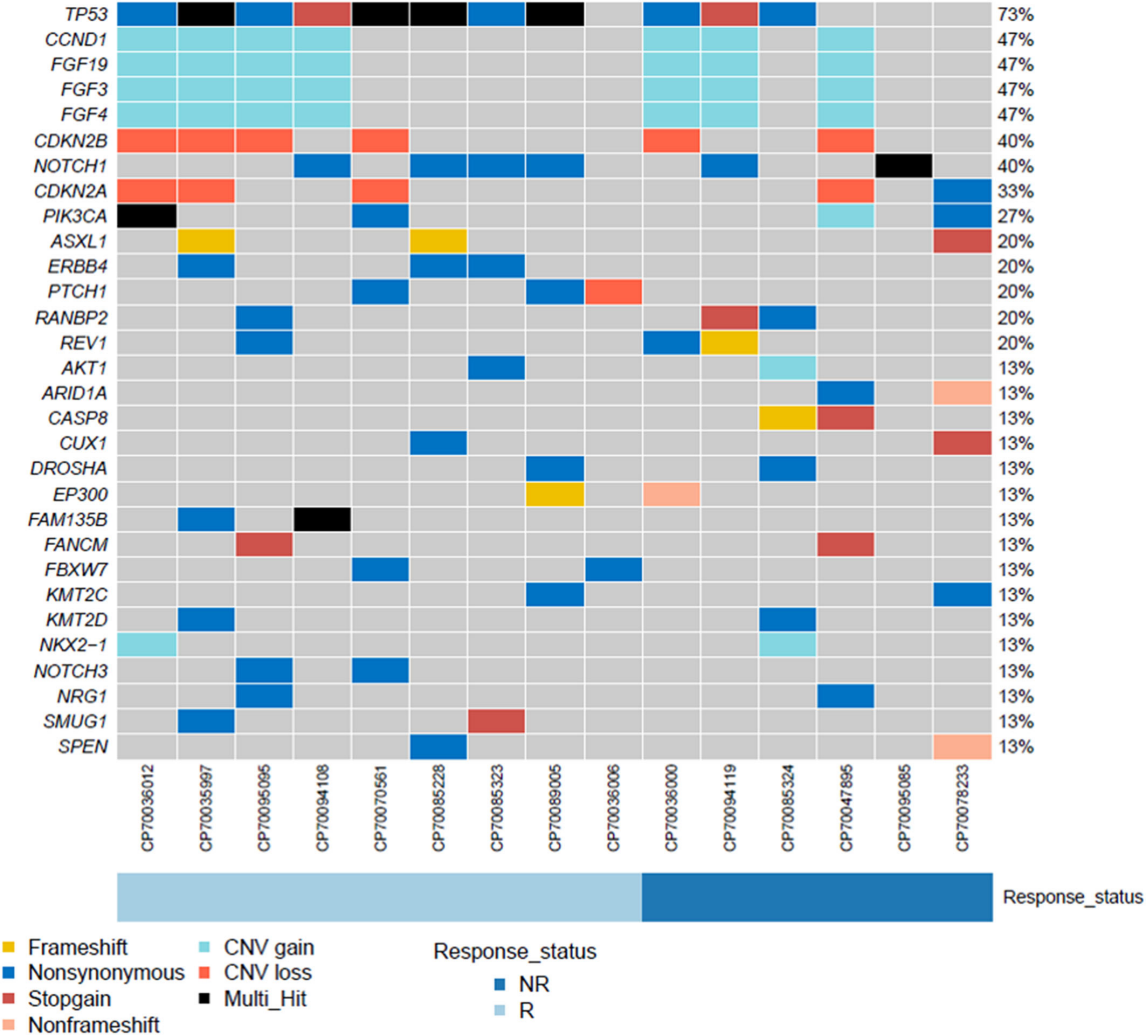
The landscape of genomic analysis conducted at the baseline in ESCC patients undergoing neoadjuvant camrelizumab combined with chemotherapy.

The next-generation sequencing analysis showed deleterious somatic variations in the combined group, including single-nucleotide variants (15/15, 100%) and copy number variations (10/15, 67%) (**Figure 6**). TP53 showed the highest mutation rate (12/15, 80%), with a proportion of 6/9 in responders and 4/6 in non-responders (p = 1.0, Fisher exact´s test). Of interest, 7 out of 15 (47%) patients reported co-mutations of CCND1, FGF19, FGF4 and FGF3. While no significant differences were found in these co-mutations genes between responders (4/9) and non-responders (3/6) (p = 1.0, Fisher exact´s test). Microsatellite stability was observed in all samples obtained from the combined group, with a mean tumor mutational burden of 7.86 ± 2.75 mutations/Mb.

### 3.6 Safety

The incidence of adverse events is reported in **Table 3**. There were mainly haematological events in the combined treatment group. The main grade 3 and 4 adverse events were haematologic toxicities in combined group and chemotherapy group (grade 3-4 leukopenia 20.0% vs 6.7%, grade 3-4 neutropenia 13.3% vs 6.7%), grade 1 and 2 adverse events occurred mainly in the combined treatment group(grade 1-2 anemia 53.3% vs 26.7%, grade 1-2 leukopenia 20.0% vs 6.7%, grade 1-2 thrombocytopenia 46.7% vs 20.0%, grade 1-2 neutropenia 13.3% vs 0%). The addition of camrelizumab to systemic chemotherapy led to an increased risk for severe leukopenia. Three events were reported in the combined group, compared with one event reported in the chemotherapy group. Considering severe neutropenia, 2 events were reported in the combined group and one event was reported in the chemotherapy group. The safety profile was similar between the two study groups in non-hematologic. There were no serious postoperative complications after surgery in either group, there were no new complication.

**Table 3:** Adverse events comparison of the two groups.

All events	No. of patients (%)			
	Camrelizumab + DCF		DCF	
	Grade 1–2	Grade 3–4	Grade 1–2	Grade 3–4
Gastrointestinal				
Nausea	3 (20.0)	0 (0)	3 (20.0)	0 (0)
Vomiting	1 (6.7)	0 (0)	1 (6.7)	0 (0)
Diarrhea	1 (6.7)	0 (0)	1 (0)	0 (0)
Constipation	0 (0)	0 (0)	0 (0)	0 (0)
Hematopoietic				
Anemia	8 (53.3)	0 (0)	4 (26.7)	0 (0)
Leukopenia	3 (20.0)	3 (20.0)	1 (6.7)	1 (6.7)
Thrombocytopenia	7 (46.7)	0 (0)	3 (20.0)	0 (0)
Neutropenia	2(13.3)	2(13.3)	0 (0)	1(6.7)
Other adverse events				
Dermatitis	0 (0)	0 (0)	0 (0)	0 (0)
Weak	1 (6.7)	0 (0)	0 (0)	0 (0)
Alopecia	5(33.3)		5(33.3)	

All adverse events were reported according to the National Cancer Institute Common Terminology Criteria for Adverse Events, version 5.0.

## 4 Discussion

Immunotherapy with immune checkpoint inhibitors has revolutionized the treatment for advanced cancers, including esophageal cancer. Camrelizumab is now used as a second-line therapy for advanced or metastatic ESCC. We found that neoadjuvant camrelizumab combined with chemotherapy may be a promising therapeutic approach for locally advanced ESCC. A phase III open label study (ESCORT) has recently determined the efficacy of camrelizumab vs. chemotherapy for advanced ESCC (15). The median overall survival for the camrelizumab group was 8.3 months, compared to 6.2 months for the chemotherapy group. Another phase II, single arm study (ESPRIT) investigated camrelizumab combined with paclitaxel and nedaplatin as neoadjuvant therapy for 23 patients with locally advanced ESCC (16). The ORR was 56.5% and the DCR was 100%. In Shen and Wu’s group, patients underwent neoadjuvant chemotherapy in combination with immunotherapy. The DCR have been reported in both researches (96.4% and 100%, respectively) (8, 17). In the current study, the R0 resection rates are in the range of 91.2% - 100% (8, 15–17). R0 resection rate is 95.4% in our in our laboratory during 2019 – 2022. Our findings were consistent with these studies. Nagai et al. investigated neoadjuvant chemotherapy and subsequent esophagectomy for 141 Asiatic patients with ESCC (18). About 7.1% of the patients showed a pathological complete response. Upon endoscopic evaluation, no response, partial response and good response were observed in 46 (32.6%), 54 (33.3%) and 41 (29.1%) of the patients, respectively. In our study, we found a discordance between imaging and pathological assessments. Inflammation may mimic tumor invasion and, therefore, lead to false positive results (19). The response rate in previous studies was slightly higher than that in our study, ranging from 53.7 to 64.3% (20). This can be due to different issues, including selection and observation bias. Consistently with Nagai et al., 6.7% (1/15) of patients in the chemotherapy group reported a pathological complete response.

Previously, Wang et al. investigated the genetic alternations of Chinese patients with ESCC (21). We conducted a gene mutation analysis using next-generation sequencing techniques. TP53 was the most common mutated gene, with a mutation rate of 87% (13/15). Wang et al. reported a slightly lower mutation rate. Among the copy number amplified genes, the 11q13 amplicon (*CCND1/FGF19/FGF4/FGF3*) showed the highest frequency (47%, 7/15). This result was consistent with previously published reports (21, 22). The *CCND1/FGF19/FGF4/FGF3* co-amplification has been previously associated with a poor prognosis (22). However, we did not find similar findings.

The tumor microenvironment is characterized by the presence of tumor cells, stromal cells and immune cells (23). The composition of the tumor microenvironment may affect the antitumor immune response (24). We investigated the dynamic changes of the tumor immune microenvironment before and after neoadjuvant therapy in both groups. Compared to chemotherapy alone, neoadjuvant camrelizumab combined with chemotherapy influenced the densities of CD3^+^, CD4^+^ and CD8^+^ T cells, M1 tumor-associated macrophages, CD20^+^ B cells and T cells expressing PD1 in the tumor microenvironment. The immune cells were more abundant in the tumor microenvironment, resulting in a more prolonged efficacy of neoadjuvant camrelizumab.

Immune cells, which are vital components of the tumor microenvironment, play a central role in immune responses to cancer (11, 25, 26). Immune biomarker can be used as predictors of immunotherapy, including when combined with chemotherapy. However, most biomarkers do not predict responses when immunotherapy is combined with chemotherapy. Yang et al. demonstrated that a higher presence of CD56dim NK cells was associated with a better pathological response (10). Jiang and colleagues previously showed that M1 macrophages in the tumor stroma are prognostic in patients with ESCC (27). In particular, patients with a high infiltration of M1 macrophages in the tumor stroma showed a better overall survival compared to patients with a low infiltration (28). In our study, we found that M1 tumor-associated macrophages and CD56dim NK cells in the tumor stroma were associated with a better response to neoadjuvant camrelizumab combined with chemotherapy. In the chemotherapy group, we did not find similar findings. The M1 tumor-associated macrophages are considered antitumor effector cells enhancing antigen-presenting ability of dendritic cells (25, 26, 28). The CD56dim NK cells are the major subset of NK cells in peripheral blood that kill cancer cells by secreting perforins and granzymes (29, 30). Yang and colleagues reported that CD56dim NK cells were significantly more abundant in responders than in non-responders when camrelizumab was combined with chemotherapy (10). However, the study was limited by the absence of a control group. We reported similar findings with the implementation of a control group, excluding the possibility that chemotherapy alone could affect the immune microenvironment.

For instance, whereas several researchs have found that TAMs generally have a detrimental impact on the prognosis of gastric patients (31–33), several investigations have found the opposite (34, 35). Most studies do not really evaluate M1 and M2 TAMs independently; instead, they solely include the total number of macrophages (M1 + M2) in their analyses. To reconcile these controversies, research has demonstrated that polarization of TAMs in cancer, especially the M1/M2 ratio, is a more physiologically meaningful indication for cancer prognosis than TAM concentrations as a whole (36–38). These studies has been suggested that patients with higher M1/M2 ratio have a more favorable prognosis. Mechanistically, a higher M1/M2 ratio promotes cytokine production of T cells (39). Therefore, the M1/M2 ratio is a crucial factor in determining a patient’s prognosis. In this study, we evaluated the M1/M2 ratio of responders and non-responders in both study groups. The M1/M2 ratio in the combined group was significantly higher in responders than in non-responders, suggesting that a higher M1/M2 ratio may enhance the tumor killing properties of infiltrating T cells. A previous *in vitro* study reported that a high M1/M2 ratio was not associated with an antineoplastic effect in CT26 tumors (40). The contrasting finding may be due to an overabundance of M2 macrophages, whose role in promoting tumor growth is dual (41, 42).

Consistent with previously published studies, the majority of reported adverse events were acceptable in both groups (15). Patients in the combined group experienced mild to moderate thrombocytopenia more often than patients in the chemotherapy group. Nausea, anemia and thrombocytopenia were the most common adverse events described in the chemotherapy group. A few severe adverse events were also reported, such as severe leukopenia and neutropenia.

This study is affected by some limitations. First limitation regards the sample size due to poor sample accessibility, which could still be considered not large enough, although other similar studies have already obtained meaningful results with even smaller sample sizes (9, 10). It is important to emphasize that this is an interim study in which the evaluation of response to neoadjuvant related M1/M2 was developed. The next step will consist of selecting a much larger sample to validate the observed results.

Secondly, most of the patients in the combined group did not undergo surgery. Thus, gastroscopic biopsy samples were evaluated instead of using the Mandard tumor regression grading system. Thirdly, we did not do a survival analysis due to insufficient survival data.

## 5 Conclusions

We showed that neoadjuvant camrelizumab combined with chemotherapy improved ORR numerically in locally advanced ESCC compared to chemotherapy alone. High baseline M1/M2 ratios in the tumor region were potentially associated with a better response to neoadjuvant camrelizumab combined with chemotherapy, implying a potential role as prognostic markers. Further studies with a larger sample size are warranted to define the M1/M2, M1 tumor associated macrophages and CD56dim NK cell values for predicting efficacy.

## Data availability statement

The raw data supporting the conclusions of this article will be made available by the authors upon reasonable request.

## Ethics statement

The studies involving human participants were reviewed and approved by Ethics Committee of Xi Jing Hospital, The Fourth Military Medical University. The patients/participants provided their written informed consent to participate in this study.

## Author contributions

JY, ShuW, GX, ML, JZ, YN put forward the content of the paper. ShuW, GX, WD and HZ wrote the manuscript. The others literature and clinical data were collected and reviewed. All authors contributed to the article and approved the submitted version.
